# *Lactococcus lactis* endocarditis and liver abscess in an immunocompetent patient: a case report and review of the literature

**DOI:** 10.1186/s13256-022-03676-1

**Published:** 2023-03-31

**Authors:** Wahib Lahlou, Abderrahim Bourial, Taib Maaouni, Ahmed Bensaad, Ilham Bensahi, Mohamed Sabry, Mohamed Miguil

**Affiliations:** 1 Department of Polyvalent Resuscitation Unit, Cheikh Khalifa International University Hospital, Mohammed VI University of Sciences and Health, Casablanca, Morocco; 2 Department of Visceral Surgery, Cheikh Khalifa International University Hospital, Mohammed VI University of Sciences and Health, Casablanca, Morocco; 3 Department of Cardiology, Cheikh Khalifa International University Hospital, Mohammed VI University of Sciences and Health, Casablanca, Morocco; 4 Cheikh Khalifa International University Hospital, Mohammed VI University of Sciences and Health, Casablanca, Morocco

**Keywords:** Endocarditis, *Lactococcus lactis*, *Lactococcus lactis* subsp. *cremoris*, Liver abscess, Splenic infarction

## Abstract

**Background:**

Over the last two decades, several cases of infections caused by *Lactococcus lactis* have been reported. This Gram-positive coccus is considered non-pathogenic for humans. However, in some rare cases, it can cause serious infections such as endocarditis, peritonitis, and intra-abdominal infections.

**Case presentation:**

A 56-year-old Moroccan patient was admitted to the hospital because of diffuse abdominal pain and fever. The patient had no past medical history. Five days before his admission, he developed abdominal pain in the right lower quadrant along with chills and feverish sensations. Investigations showed a liver abscess, which was drained, and the microbiological study of the pus revealed *Lactococcus lactis* subsp. *cremoris*. Three days after admission, control computed tomography objectified splenic infarctions. Cardiac explorations were performed and showed a floating vegetation on the ventricle side of the aortic valve. We retained the diagnosis of infectious endocarditis according to the modified Duke criteria. The patient was declared afebrile on day 5 and the evolution was clinically and biologically favorable. *Lactococcus lactis* subsp. *cremoris*, formerly known as *Streptococcus* *cremoris*, is a rare cause of human infections. The first case of *Lactococcus lactis* *cremoris* endocarditis was reported in 1955. This organism includes three subspecies: *lactis*, *cremoris*, and *hordniae*. A MEDLINE and Scopus search showed only 13 cases of infectious endocarditis due to *Lactococcus lactis*, with subsp. *cremoris* identified in four of the cases.

**Conclusions:**

To our knowledge, this is the first case report of the co-occurrence of *Lactococcus lactis* endocarditis and liver abscess. Despite its reported low virulence and good response to antibiotic treatment, *Lactococcus lactis* endocarditis must be considered a serious disease. It is imperative for a clinician to suspect this microorganism of causing endocarditis when they notice signs of infectious endocarditis in a patient with a history of consumption of unpasteurized dairy products or contact with farm animals. The finding of a liver abscess should lead to an investigation of endocarditis, even in previously healthy patients without obvious clinical signs of endocarditis.

## Background

Over the last two decades, several cases of infections caused by *Lactococcus lactis* have been reported. Widely used for the production of fermented products, this Gram-positive coccus is considered nonpathogenic for humans [1]. However, in some rare cases involving both immunocompetent and immunocompromised patients [2], it can cause serious infections such as endocarditis, peritonitis, and intra-abdominal infections[3].

In this article, we report a rare case of an immunocompetent 56-year-old male with a history of raw milk consumption who presented with a *Lactococcus*-associated liver abscess and endocarditis.

To our knowledge, this is the first reported case in the literature (on the basis of the MEDLINE and Scopus databases) of a concurrent discovery of the association of a liver abscess and an endocarditis caused by *Lactococcus lactis*.

## Case presentation

A 56-year-old Moroccan patient was admitted to the hospital for diffuse abdominal pain and fever. The patient was a bricklayer, reported no past medical history and no alcohol consumption, but was an active smoker. Five days before admission, the patient developed abdominal pain in the right lower quadrant along with chills and feverish sensations. Initially, the patient received symptomatic treatment with phloroglucinol (80 mg three times a day) and showed no improvement. In contrast, his abdominal pain increased and became diffuse.

On admission, his temperature was 39 °C, his pulse was 126 beats per minute, his blood pressure was 140/76 mmHg, his respiratory rate was 15 breaths per minute, his oxygen saturation was 94% in ambient air, and his Glasgow Coma Scale (GCS) score was 15/15.

Physical examination showed diffuse abdominal tenderness on palpation and decreased vesicular murmurs in the right chest. There were no valvular murmurs, and no mucocutaneous lesions of infectious endocarditis such as Janeway lesions, Osler nodes, or splinter hemorrhages under the fingernails. The rest of the examination was normal.

The biological assessment, carried out in the emergency department, showed leucocytosis with neutrophilia, hypereosinophilia, and monocytosis (white blood cell count of 14,960 per mm^3^, neutrophil count of 9930 per mm^3^, eosinophil count of 2570 per mm^3^, and monocyte count of 1210 per mm^3^). The plasma C-reactive protein level was 419.37 mg/l, and the procalcitonin level was 4.660 ng/ml. The liver function tests were elevated, with an aspartate aminotransferase level of 139.1 UI/L and an alanine aminotransferase level of 279 UI/L. Kidney function was normal with a creatinine level of 8.7 mg/L and urea level of 0.52 g/L. The electrocardiogram performed on admission was normal.

**Fig. 1:**
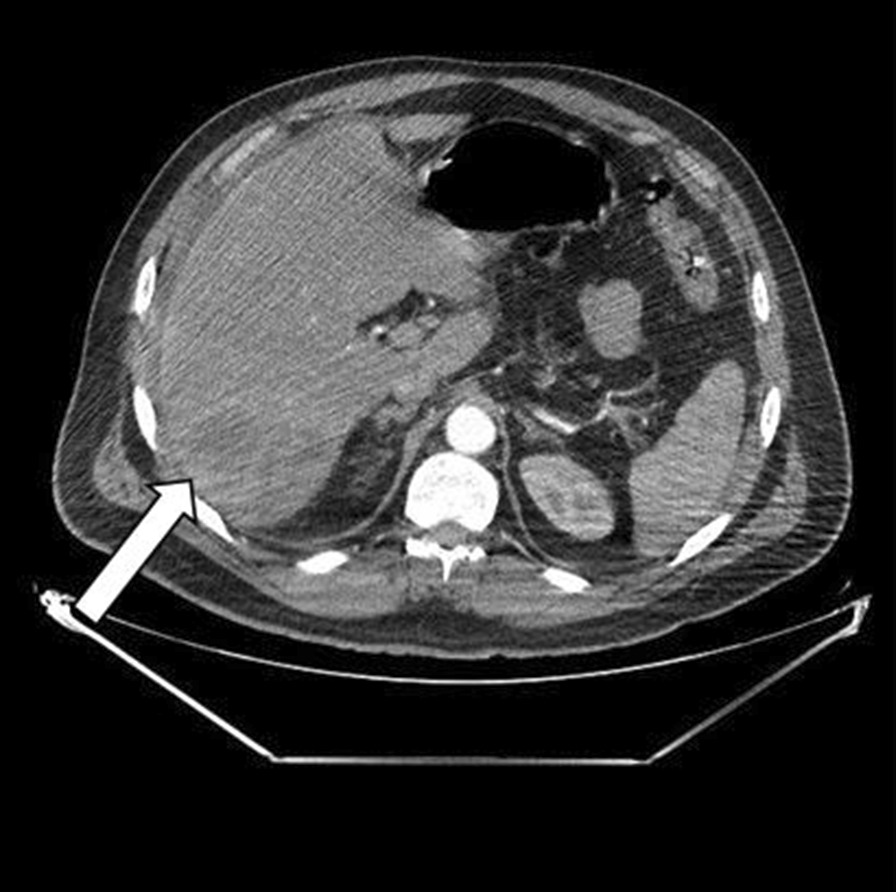
Abdominal computed tomography revealed a multiloculated hypodensity in segment VII, suggesting a liver abscess (white arrowhead)

**Fig. 2:**
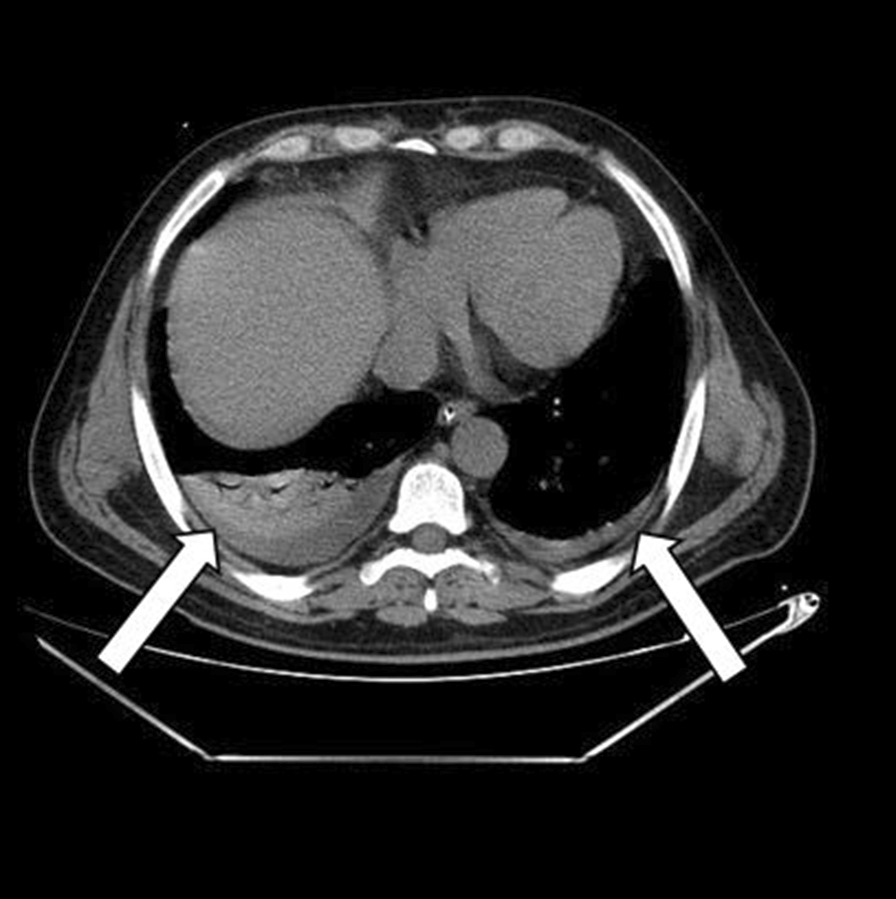
Thoracic computed tomography revealed a mild predominantly right bilateral pleural effusion (white arrowheads)

Thoraco-abdomino-pelvic computed tomography (CT) showed right basal pneumonia, mild bilateral pleural effusion, low abundance perihepatic fluid effusion, and an abscess in segment VII of the liver measuring 87 × 70 mm. (Figs. 1 and 2).

Surgical drainage was recommended and performed under general anesthesia. The exploration showed a fissured abscess through which we noted the exit of pus. It was drained by a Salem sump drain with pus sampling for cytobacteriological study. The pus was cultured and grew *Lactococcus lactis* subsp. *cremoris*. During hospitalization, repeated aerobic and anaerobic blood cultures were taken and cultured, all of which were negative.

**Fig. 3:**
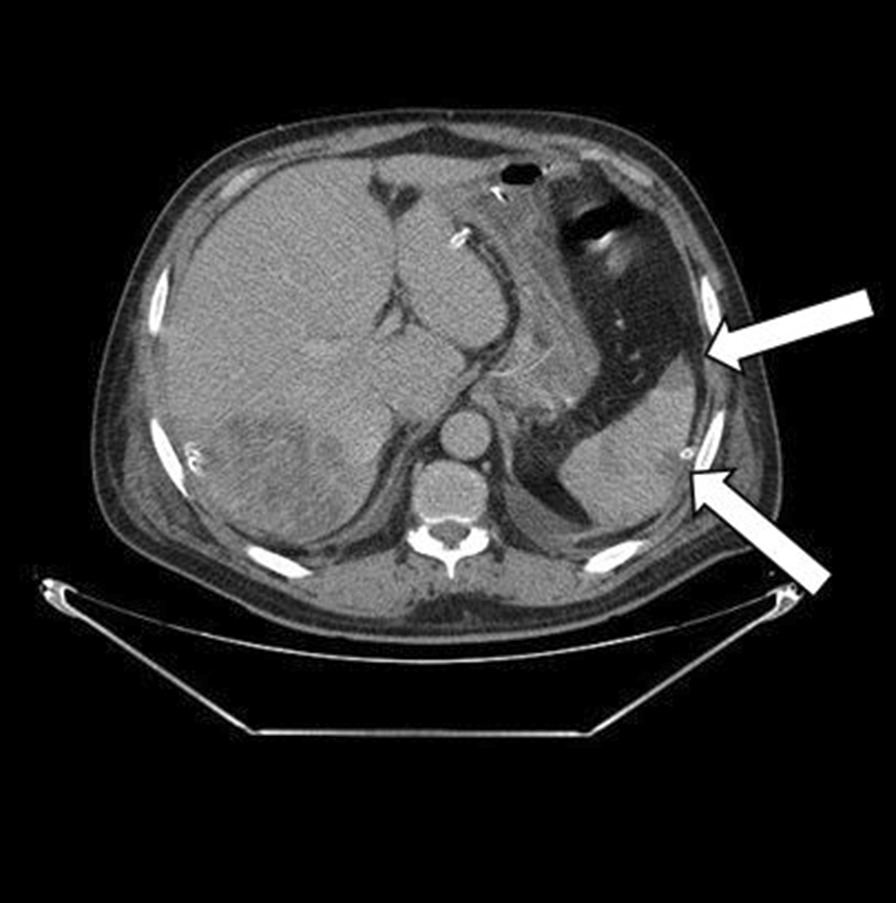
Control abdominal computed tomography revealed areas of splenic infarction (white arrowheads)

**Fig. 4:**
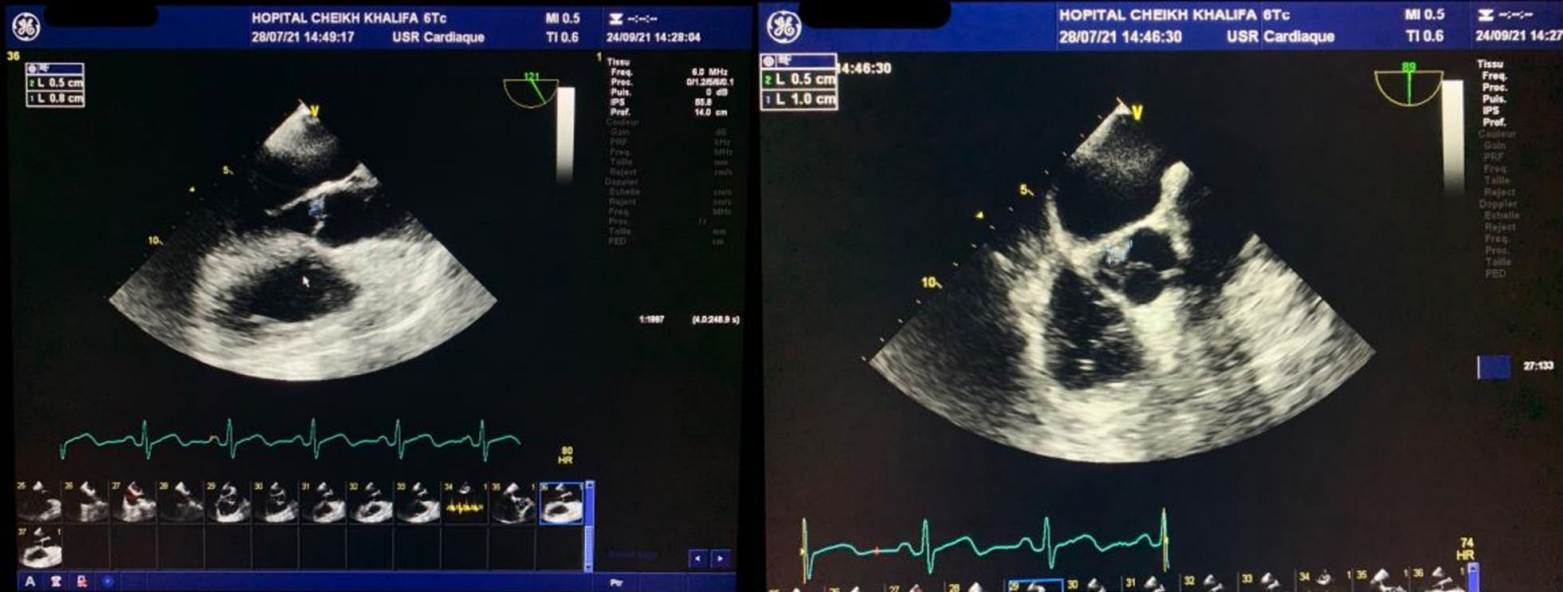
Transesophageal echocardiography showed a floating and vibratile vegetation measuring 8 × 5 mm on the ventricular side of the aortic valve

The postoperative follow-up was simple. The patient was treated empirically with intravenous antibiotics: metronidazole (500 mg three times daily), imipenem (500 mg four times daily), and amikacin (1 g once a day). The amikacin was stopped at day 5. Three days after admission, we carried out a control CT that objectified a more organized aspect of the pre-suppurative phase liver abscesses and areas of splenic infarction (Fig. 3). Transthoracic echocardiography was performed and was normal with no evidence of vegetations, no valvular or paravalvular regurgitation, and no aortic abscess or pericardial effusion. A decision to carry out transesophageal echocardiography was made, where a floating vegetation on the ventricle side of the aortic valve measuring 8 × 5 mm was observed and there were no imaging of an abscess or Valsalva aneurysm (Fig. 4).

Investigations of immune function demonstrated that he was HIV negative.

The patient was declared afebrile on day 5. The drainage catheter was removed on day 6 when the drained fluid was minimal.

As part of the endocarditis extension assessment, the patient underwent a cerebral CT that showed no abnormalities.

In total, the patient was treated with imipenem for 4 weeks and metronidazole was also continued for 3 weeks to cover other potential anaerobic germs.

The outcome was clinically and biologically favorable with negativation of the infection’s biomarkers (procalcitonin, C-reactive protein, and total white blood cell count) without occurrence of AV-type electrical complications.

**Fig. 5:**
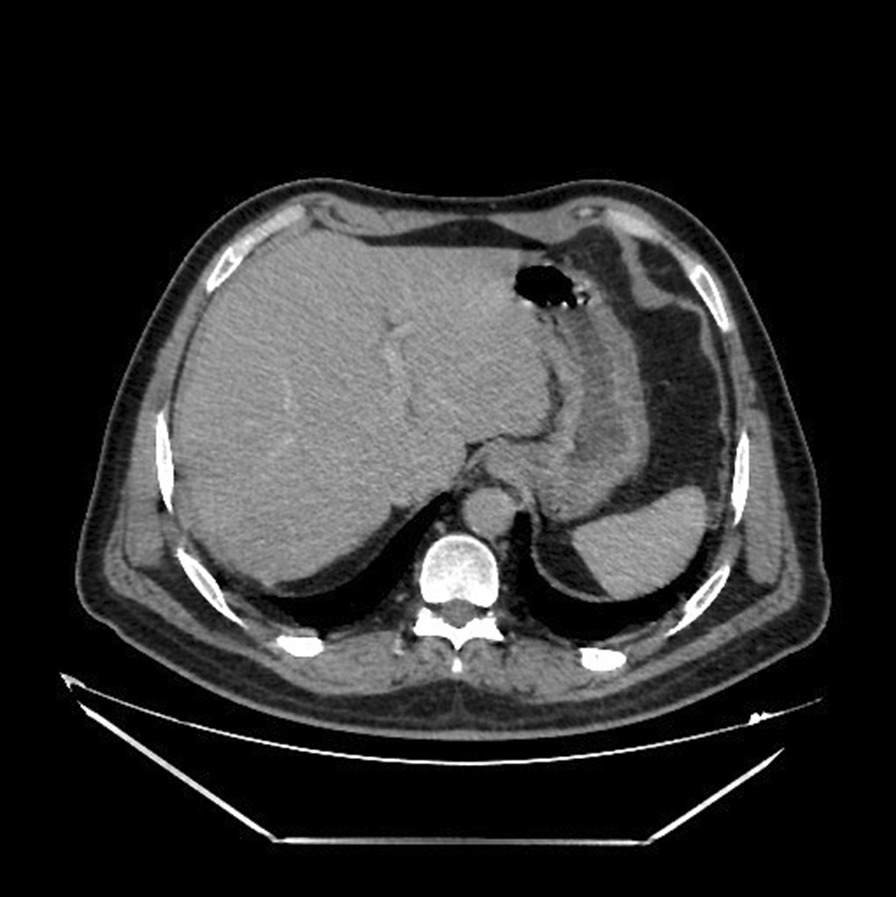
Control computed tomography on follow-up after 2 months showed almost complete regression of the liver abscess and splenic infarctions

On follow-up in the outpatient clinic after 2 months, the patient was well and asymptomatic with normalized biochemistry and almost complete regression of the liver abscess and splenic infarctions on CT (Fig. 5). Moreover, on the control transesophageal echocardiography, we remarked that the vegetation had decreased in size without the occurrence of any complications.

The patient was reviewed in consultation after 8 months, and was totally asymptomatic.

## Discussion and conclusions

To our knowledge, and up to date in the current literature, this is the first described case of concurrent discovery of a liver abscess associated with endocarditis caused by *Lactococcus lactis.*

*Lactococcus lactis* is a Gram-positive bacterium used for the production of fermented dairy products, particularly cheddar cheese. Five species of the genus Lactococcus have been described: *L. lactis*, *L. garvieae*, *L. piscium*, *L. plantarum*, and *L. raffinolactis*[4, 5].

*L. lactis* actually includes three subspecies: *L. lactis* subsp.* lactis*, *L. lactis* subsp.* cremoris*, and *L. lactis* subsp.* hordniae* [4, 5].

Most bacteria employed in food preparation are killed during digestion (following ingestion), but it has been established that *Lactococcus* remains viable after transit through the gastrointestinal tract, which is considered to be the mechanism of *Lactococcus lactis* infection in humans, especially when there is a loss of intestinal wall integrity[2, 6]. This may justify colonoscopy as part of the investigation.

In some cases (similar to ours), the ingestion of unpasteurized milk, sour cream, or yogurt was recognized. However, in other cases, there was no history of ingestion of unpasteurized dairy products. Owing to the rarity of *Lactococcus lactis* infection, the source of this infection has not been well demonstrated. The hypotheses regarding the source of infection include the ingestion of unpasteurized dairy products or direct intraluminal inoculation from contaminated hands[7].

*Lactococcus lactis* has low virulence and is considered nonpathogenic. However, it has recently been considered an opportunistic pathogen microbe. A pathophysiological mechanism has been described to explain the virulence of this microorganism in the genesis of infectious endocarditis. Indeed, a study carried out in 2016 showed that *Lactococcus lactis* expresses the glycoprotein Cnm, which promotes its adhesion to type I collagen and to cardiac tissues (in particular the tissues of the aortic valve) [8].

We retained the diagnosis of infectious endocarditis for our patient according to the modified Duke criteria. [9, 10]. Repeated blood cultures taken during hospitalization did not grow any specific pathogen that could probably be attributed to previous antibiotic treatment. According to the literature, the rate of culture-negative endocarditis varies from 2.1% to 35% [11]. In our case, the vegetation was not visualized on transthoracic echocardiography although it was a quite voluminous aortic localization and this imaging had good sensitivity for the exploration of the aortic sigmoids [12]. This highlights the fact that it is necessary to perform transesophageal echocardiography when endocarditis is a clinical possibility.

Infectious endocarditis due to *Lactococcus lactis* is very rare, and a MEDLINE and Scopus search (Table 1) identified only 13 cases of infectious endocarditis due to *Lactococcus lactis*, with the subsp. *cremoris* identified in four of the cases. In these four cases, including ours, the affected valve was the aortic valve. However, in other cases, endocarditis affected the mitral valve, which is the most commonly involved valve, followed by the tricuspid valve. The outcomes of the few reported cases were good: only two patients died (including one infant diagnosed postmortem and one adult whose course was complicated by bilateral uncal herniation due to multiple intracerebral hemorrhages). This underlines the good prognosis of *Lactococcus lactis* endocarditis.

**Table 1:** Reported cases (MEDLINE and Scopus Databases) of infectious endocarditis caused by *Lactococcus lactis*

Authors	Year	Subspecies	Unpasteurized dairy products consumption	Heart disease	Valve involved	Complications	Outcome
Wood *et al*. [13]	1955	*L. lactis* subsp. *lactis*	Yes (ice cream)	No history of heart disease	Unknown	None	Recovered
Mannion and Rothburn [14]	1990	*L. lactis* subsp. *lactis*	Unknown	Myocardial infarction, rheumatic mitral valve disease	Mitral	Infarction/dysphasia	Recovered
Pellizzer *et al*. [15]	1996	*L. lactis* subsp. *cremoris*	No	Mitral prolapse	Aortic	None	Recovered
Halldorsdottir *et al*. [16]	2002	*L. lactis* subsp. *cremoris*	Yes (milk)	No history of heart disease	Mitral	None	Recovered
Kiss *et al*. [17]	2005	Unknown	Unknown	Unknown	Unknown	Femoral osteomyelitis	Unknown
Zechini *et al*. [18]	2006	*L. lactis* subsp. *lactis*	Unknown	Atrial mixoma, mitral regurgitation	Mitral	None	Recovered after surgery
Resch *et al*. [19]	2006	*L. lactis* subsp. *cremoris*	Yes (cheese)	No history of heart disease	Aortic	Multiple mycotic aneurysms	Recovered after surgery
Lin *et al*. [20]	2009	*L. lactis* subsp. *cremoris*	No	No heart disease	Mitral	Intracerebral hemorrhage/infraction	Deceased
Rostagno *et al*. [6]	2012	Unknown	No	Mitral valve prolapse	Mitral	Embolic infarction	Recovered after surgery
Taniguchi *et al*. [21]	2015	Unknown	No	No heart disease	Mitral + tricuspid	Arrhythmia	Deceased
Mansour *et al*. [22]	2016	Unknown	No	Ventricular septal defect	Tricuspid	Pulmonary septic emboli	Recovered
Georgountzos *et al*. [7]	2017	Unknown	No	No history of heart disease	Aortic	None	Recovered
Fei Chen *et al*. [23]	2018	*L. lactis* subsp. *lactis*	Unknown	Coronary heart disease	Mitral	None	Recovered
Lahlou *et al*.	2021	*L. lactis* subsp. *cremoris*	Yes (milk)	No heart disease	Aortic	Liver abscess, splenic infarction, bilateral pleural effusion + right basal pneumonia	Recovered

Liver abscess is the second most common localization of *Lactococcus lactis* infections after endocarditis. Reviewing the MEDLINE and Scopus databases, we found nine cases of hepatic abscess due to *Lactococcus lactis*: most frequently *Lactococcus lactis* subsp. *cremoris* in four cases, *Lactococcus lactis* subsp. *lactis* in one case, and no available data regarding the subspecies in four cases [1, 24–31].

We remark that six out of the nine cases did not accomplish proper cardiac exploration (transthoracic echocardiography nor transesophageal echocardiography). Note that in the other three cases, no available data were found.

Our case is the first liver abscess described in the literature with a proper cardiac exploration that found a co-occurrence of infectious endocarditis. We therefore wonder if this association is underdiagnosed as opposed to what is described, and find it logical to recommend systematic ultrasound cardiac exploration in the case of any *Lactococcus lactis* hepatic abscess.

A liver abscess formation is explained by its anatomical and physiological singularity. The liver receives blood from the systemic and portal circulations, from which the infected bloodstream can carry the bacterium, and the infection spread in this case was considered hematogenous. The usual pathophysiology behind liver abscess formation can be assumed as bowel content leakage and peritonitis rout. The bacteria can travel through the portal vein to reach the liver and reside there. Less frequently, the infection can originate from the biliary system[32].

Table 2 presents all available multiple localizations of *Lactococcus lactis* infections. Nine cases have been described in the literature, from which we find four cases of endocarditis and five liver abscesses as the starting point of the systemic infection.

**Table 2:** Reported cases (MEDLINE and Scopus Databases) of multifocal abscess localizations caused by *Lactococcus lactis*

References	Year	Subspecies	Unpasteurized dairy products consumption	Original infection site	Valve involved (when endocarditis is present)	Complications	Outcome
Nakarai *et al*. [24]	2000	*L. lactis* subsp. *cremoris*	No	Liver abscess	No cardiac assessment was performed	Massive right pleural effusion and collapse of the right lower lobe	Recovered
Antolín *et al*. [1]	2004	*L. lactis* subsp. *cremoris*	No	Diverticulitis that lead to liver abscess	No cardiac assessment was performed	Massive right pleural effusion	Recovered
Kiss *et al*. [17]	2005	Unknown	Unknown	Endocarditis	Unknown	Femoral osteomyelitis, cerebral, and pulmonary abscess	Unknown
Resch *et al*. [19]	2006	*L. lactis* subsp. *cremoris*	Yes (cheese)	Endocarditis	Aortic	Epididymitis, Reiter’s syndrome, multiple mycotic aneurysms, eye and kidney embolii	Recovered
Lin *et al*. [20]	2009	*L. lactis* subsp. *cremoris*	No	Endocarditis	Mitral	Intracerebral hemorrhage	Deceased
Kim *et al*. [28]	2010	*L. lactis* subsp. *cremoris*	No	Liver abscess and empyema and necrotizing pneumonia	No cardiac assessment was performed	Bilateral pleural effusion	Recovered
Fragkiadakis *et al*. [30]	2016	*L. lactis* subsp. *cremoris*	Yes	Bowell wall thickening, liver abscess and perirenal abscess	–	Perirenal abscess, severe periodontitis	Recovered
Mansour *et al*. [22]	2016	Unknown	Yes (cheese)	Endocarditis	Tricuspid	Pulmonary septic emboli	Recovered
Shimizu *et al*. [3]	2019	Unknown	No	Cholangitis	No cardiac assessment was performed	Liver abscesses	Deceased
Lahlou *et al*.	2021	*L. lactis* subsp. *cremoris*	Yes (milk)	Liver abscess	Aortic	Bilateral pleural effusion + right basal pneumonia	Recovered

Six out of the nine cases indicated *Lactococcus lactis* subsp. *cremoris*, knowing that the other three cases lacked information and did not specify the subspecies of *Lactococcus lactis*. Therefore, the *cremoris* subspecies is more likely to be spread systemically and carried to multiple organs, regardless of the infection starting point, as this hypothesis is consistent with our case.

We found that consumption of unpasteurized milk or cheese was present in three out of the nine cases. It is plausible that the inoculation of the microorganism occurred through an orofecal mechanism. Although *Lactococcus* inoculation is more prone to be accomplished through the digestive duct, endocarditis and liver abscess seem to be equally frequent as a triggering systemic infection site.

Underlying conditions were documented in eight out of the nine cases identified in the MEDLINE and Scopus database articles and only one patient had an immunocompromised state related to his condition (cholangiocarcinoma). On the basis of the literature, immunodeficiency or immunocompromisation may be a predisposing factor linked with *Lactococcus lactis* infection with liver abscess localization[3].

The most frequent localizations that lead to multiple localizations infection other than the heart and the hepatobiliary tract are the pleura, lungs, brain, retroperitoneal organs, bones, and so on, as they are often the starting infection sites. [3]

The majority of hepatobiliary-starting *Lactococcus* infection cases did not benefit from proper cardiac exploration. We found that cases 2, 6, and 9 had positive blood culture with a non-specific endocarditis germ (minor modified Duke criteria) with some other minor criterion. On the basis of the modified Duke criteria, these cases had a score of 3, compatible with a possible endocarditis diagnosis. We therefore propose a systematic ultrasound cardiac exploration in front of any *Lactococcus lactis* hepatic abscess or multiple localization *Lactococcus lactis* infection, looking for an endocarditis echocardiographic finding.

Pleural effusion, especially in the right chest, was frequently linked with *Lactococcus lactis* infections, particularly when the infection started with liver abscesses. The possible mechanism of propagation might be through the bloodstream, as well as through a locoregional process, considering the anatomical links.

The outcome was favorable in the majority of cases, suggesting that early treatment may be a major prognostic factor in this kind of infection.

One of the interesting findings in this case was the elevated monocyte count. This patient presented concomitant monocytosis within the endocarditis episode. It is known that monocytosis can be caused by a wide variety of neoplastic and nonneoplastic conditions [33]. The various causes of monocytosis can be divided into two broad categories: clonal or reactive, from which acute infections such as endocarditis can be an etiologic factor [34]. Infective-endocarditis-related bacteria are described in the literature as activating factors of blood monocytes. Activated monocytes are believed to be important factors, participating in the formation of endocarditis vegetations by producing cytokines and procoagulant factors that enhance the development of the infected coagulum, formally known as vegetation [35, 36].

*Lactococcus lactis*, formerly known to be a nonpathogenic microorganism, has contributed to several cases of human infections, including infectious endocarditis and liver abscesses. It is essential for a clinician to suspect this microorganism of causing endocarditis when they notice signs of infectious endocarditis in a patient with a history of consumption of unpasteurized dairy products or contact with farm animals. This case suggests the hypothesis that the finding of a liver abscess may require an investigation of endocarditis even in previously healthy patients without obvious clinical signs of endocarditis.

Here, we report here the fourteenth case of endocarditis caused by *Lactococcus lactis* described in the literature and the first case report of the co-occurrence of *Lactococcus lactis* endocarditis and liver abscess.

## Data Availability

The datasets used and/or analyzed during the current study are available from the corresponding author on reasonable request.

## Abbreviations

CT: Computed tomography
HIV: Human immunodeficiency virus
*L. lactis*: *Lactococcus lactis*
*L. garvieae*: *Lactococcus garvieae*
*L. piscium*: *Lactococcus piscium*
*L. plantarum*: *Lactococcus plantarum*
*L. raffinolactis*: *Lactococcus rafinolactis*

## References

[1] Antolín J, Cigüenza R, Salueña I, Vázquez E, Hernández J, Espinós D (2004). Liver abscess caused by *Lactococcus lactis* cremoris: a new pathogen. Scand J Infect Dis.

[2] Drouault S, Corthier G, Ehrlich SD, Renault P (1999). Survival, physiology, and lysis of *Lactococcus lactis* in the digestive tract. Appl Environ Microbiol.

[3] Shimizu A, Hase R, Suzuki D, Toguchi A, Otsuka Y, Hirata N (2019). Lactococcus lactis cholangitis and bacteremia identified by MALDI-TOF mass spectrometry: a case report and review of the literature on *Lactococcus lactis* infection. J Infect Chemother.

[4] Song AA-L, In LLA, Lim SHE, Rahim RA (2017). A review on *Lactococcus lactis*: from food to factory. Microb Cell Factories..

[5] Parapouli M, Delbès-Paus C, Kakouri A, Koukkou A-I, Montel M-C, Samelis J (2013). Characterization of a wild, novel nisin A-producing Lactococcus strain with an *L. lactis* subsp. cremoris genotype and an *L. lactis* subsp. lactis phenotype, isolated from Greek raw milk. Appl Environ Microbiol..

[6] Rostagno C, Pecile P, Stefàno PL (2013). Early *Lactococcus lactis* endocarditis after mitral valve repair: a case report and literature review. Infection.

[7] Georgountzos G, Michopoulos C, Grivokostopoulos C, Kolosaka M, Vlassopoulou N, Lekkou A (2018). Infective endocarditis in a young adult due to *Lactococcus lactis*: a case report and review of the literature. Case Rep Med.

[8] Freires IA, Avilés-Reyes A, Kitten T, Simpson-Haidaris PJ, Swartz M, Knight PA (2017). Heterologous expression of Streptococcus mutans Cnm in *Lactococcus lactis* promotes intracellular invasion, adhesion to human cardiac tissues and virulence. Virulence.

[9] Habib G, Lancellotti P, Antunes MJ, Bongiorni MG, Casalta J-P, Del Zotti F (2015). 2015 ESC Guidelines for the management of infective endocarditis: the Task Force for the Management of Infective Endocarditis of the European Society of Cardiology (ESC) Endorsed by: European Association for Cardio-Thoracic Surgery (EACTS), the European Association of Nuclear Medicine (EANM). Eur Heart J.

[10] Li JS, Sexton DJ, Mick N, Nettles R, Fowler VG, Ryan T (2000). Proposed modifications to the Duke criteria for the diagnosis of infective endocarditis. Clin Infect Dis.

[11] Subedi S, Jennings Z, Chen SC-A (2017). Laboratory approach to the diagnosis of culture-negative infective endocarditis. Heart Lung Circ.

[12] Reynolds HR, Jagen MA, Tunick PA, Kronzon I (2003). Sensitivity of transthoracic versus transesophageal echocardiography for the detection of native valve vegetations in the modern era. J Am Soc Echocardiogr.

[13] Wood HF, Jacobs K, McCarty M (1955). Streptococcus lactis isolated from a patient with subacute bacterial endocarditis. Am J Med.

[14] Mannion PT, Rothburn MM (1990). Diagnosis of bacterial endocarditis caused by Streptococcus lactis and assisted by immunoblotting of serum antibodies. J Infect.

[15] Pellizzer G, Benedetti P, Biavasco F, Manfrin V, Franzetti M, Scagnelli M (1996). Bacterial endocarditis due to *Lactococcus lactis* subsp. cremoris: case report. Clin Microbiol Infect..

[16] Halldórsdóttir HD, Haraldsdóttir V, Böđvarsson Á, Þorgeirsson G, Kristjánsson M (2002). Endocarditis caused by Lactococcus cremoris. Scand J Infect Dis.

[17] Kiss J, Zahár Á, Nyíri P, Prinz G (2005). A case of femoral osteomyelitis caused by Lactococcus. Orv Hetil.

[18] Zechini B, Cipriani P, Papadopoulou S, Di Nucci G, Petrucca A, Teggi A (2006). Endocarditis caused by *Lactococcus lactis* subsp. lactis in a patient with atrial myxoma: a case report. Diagn Microbiol Infect Dis..

[19] Resch M, Schichtl T, Endemann DH, Griese DP, Kasprzak P, Djavidani B (2008). General aneurysmatosis due to cheese consumption: complications of an endocarditis caused by Lactococcus cremoris. Int J Cardiol.

[20] Lin K-H, Sy CL, Chen C-S, Lee C-H, Lin Y-T, Li J-Y (2010). Infective endocarditis complicated by intracerebral hemorrhage due to *Lactococcus lactis* subsp. cremoris. Infection.

[21] Taniguchi K, Nakayama M, Nakahira K, Nakura Y, Kanagawa N, Yanagihara I (2016). Sudden infant death due to Lactococcal infective endocarditis. Leg Med.

[22] Mansour B, Habib A, Asli N, Geffen Y, Miron D, Elias N (2016). A case of infective endocarditis and pulmonary septic emboli caused by *Lactococcus lactis*. Case Rep Pediatr.

[23] Chen F, Zhang Z, Chen J (2018). Infective endocarditis caused by *Lactococcus lactis* subsp. lactis and *Pediococcus*
*pentosaceus*: a case report and literature review. Medicine (Baltimore).

[24] Nakarai T, Morita K, Nojiri Y, Nei J, Kawamori Y (2000). Liver abscess due to *Lactococcus lactis* cremoris. Pediatr Int.

[25] Güz G, Ye ZA. Ailevi Akdeniz Atefli olan bir vakada Lactococcus Lactis’e ikincil portal ven trombozu ve karaci¤er absesi. :4.

[26] Denholm J, Horne K, McMahon J, Grayson ML, Johnson P (2006). Yoghurt consumption and damaged colonic mucosa: a case of *Lactococcus lactis* liver abscess in an immunocompetent patient. Scand J Infect Dis.

[27] Imai K, Beppu T, Hayashi H, Masuda T, Mizumoto T, Ishiko T (2007). Two cases of panperitonitis due to intraperitoneal rupture of gas-containing pyogenic liver abscesses. Jpn J Gastroenterol Surg.

[28] Kim HS, Park DW, Youn YK, Jo YM, Kim JY, Song JY (2010). Liver abscess and empyema due to *Lactococcus lactis* cremoris. J Korean Med Sci.

[29] Lee JY, Seo MY, Yang J, Kim K, Chang H, Kim SC (2014). Polymicrobial peritonitis with *Lactococcus lactis* in a peritoneal dialysis patient. Chonnam Med J.

[30] Fragkiadakis K, Ioannou P, Barbounakis E, Samonis G (2017). Intra-abdominal abscesses by *Lactococcus lactis* ssp. cremoris in an immunocompetent adult with severe periodontitis and pernicious anemia. IDCases.

[31] El Hattabi K, Bouali M, Sylvestre K, Bensardi FZ, El Bakouri A, Khalid Z (2021). Lactococcus lactis ssp lactis a rare cause of liver abscesses: a case report and literature review. Int J Surg Case Rep.

[32] Akhondi H, Sabih DE. Liver abscess. In: StatPearls. Treasure Island (FL): StatPearls Publishing; 2021. 30855818

[33] Lynch DT, Hall J, Foucar K (2018). How I investigate monocytosis. Int J Lab Hematol.

[34] Mangaonkar AA, Tande AJ, Bekele DI (2021). Differential diagnosis and workup of monocytosis: a systematic approach to a common hematologic finding. Curr Hematol Malig Rep.

[35] Veltrop MHAM, Bancsi MJLMF, Bertina RM, Thompson J (2000). Role of monocytes in experimental *Staphylococcus*
*aureus* endocarditis. Infect Immun.

[36] Werdan K, Dietz S, Löffler B, Niemann S, Bushnaq H, Silber R-E (2014). Mechanisms of infective endocarditis: pathogen–host interaction and risk states. Nat Rev Cardiol.

